# Getting on the Same Page: Consolidating Terminology to Facilitate Cross-Disciplinary Health-Related Blast Research

**DOI:** 10.3389/fneur.2021.695496

**Published:** 2021-06-24

**Authors:** Jennifer N. Belding, Michael Egnoto, Robyn M. Englert, Shannon Fitzmaurice, Cynthia J. Thomsen

**Affiliations:** ^1^Leidos, San Diego, CA, United States; ^2^Health and Behavioral Sciences Department, Naval Health Research Center, San Diego, CA, United States; ^3^Study of Terrorism and Responses to Terrorism, University of Maryland, College Park, MD, United States

**Keywords:** blast, TBI, low-level blast, explosives, overpressure, injury, military

## Abstract

The consequences of blast exposure (including both high-level and low-level blast) have been a focal point of military interest and research for years. Recent mandates from Congress (e.g., National Defense Authorization Act for Fiscal Year 2018, section 734) have further accelerated these efforts, facilitating collaborations between research teams from a variety of disciplinary backgrounds. Based on findings from a recent scoping review, we argue that the scientific field of blast research is plagued by inconsistencies in both conceptualization of relevant constructs and terminology used to describe them. These issues hamper our ability to interpret study methods and findings, hinder efforts to integrate findings across studies to reach scientific consensus, and increase the likelihood of redundant efforts. We argue that multidisciplinary experts in this field require a universal language and clear, standardized terminology to further advance the important work of examining the effects of blast exposure on human health, performance, and well-being. To this end, we present a summary of descriptive conventions regarding the language scientists currently use when discussing blast-related exposures and outcomes based on findings from a recent scoping review. We then provide prescriptive conventions about how these terms should be used by clearly conceptualizing and explicitly defining relevant constructs. Specifically, we summarize essential concepts relevant to the study of blast, precisely distinguish between high-level blast and low-level blast, and discuss how the terms acute, chronic, exposure, and outcome should be used when referring to the health-related consequences of blast exposure.

## Introduction

Despite advances in combat casualty care and personal protective equipment, blast exposure remains a leading cause of morbidity and mortality for members of the U.S. Armed Forces deployed to combat environments (1, 2). For example, service members in close proximity to an improvised explosive device (IED) may, upon detonation, be killed or subject to a range of injuries, including those to their extremities (e.g., traumatic amputations), brain (e.g., traumatic brain injury), various organ systems (e.g., blast lung injury), mental health (e.g., posttraumatic stress disorder), and much more (3). To understand the implications of blast exposure for human health, experts from a plethora of scientific disciplines, including physics, engineering, and medicine, must work together effectively to generate findings that have the potential to inform blast injury prevention, mitigation, and treatment efforts (4). Although research on the health consequences of high-level blast (HLB) and low-level blast (LLB) exposure has grown rapidly over time (5), it remains in its infancy.

In general, it can be challenging for scientists from disparate fields to work together effectively and to stay abreast of published findings in other disciplines (6). When research crosses disciplinary boundaries or moves from preclinical to clinical research, terminology issues can also result from discipline-specific terms that may not be clearly understood by a wide variety of audiences. In the case of blast research specifically, efforts are often hampered by disagreement about relevant concepts and inconsistent use of terminology. For example, although research suggests there are extensive differences in the nature of the blasts to which service members may be exposed, little published research has clearly defined relevant terms or articulated the critical elements required to adequately describe or quantify these differences. Additionally, there is currently no widely accessible document that describes what these terms and concepts mean for professionals from varying disciplines. For example, neurologists who treats patients recovering from blast-induced traumatic brain injury may not understand the difference between incident and reflective overpressure (terms that we subsequently clarify), which may hinder their ability to understand findings from animal research and their application to humans. Similarly, engineers may not understand differences between acute and chronic injury, which may make it challenging for them to understand the variety of potential effects of blast exposure on long-term health. That these terms may also differ for those conducting preclinical vs. clinical research adds further challenges.

When scientists are unable to identify and address the same constructs due to the use of inconsistent or vague terminology, redundant and fractured literatures develop, which slows the advancement and spread of knowledge and serves as a barrier to effective cross-disciplinary collaboration (7). Therefore, it is imperative that we begin to work together toward a cohesive multidisciplinary research framework using a universal language that is clear to scientists from a wide variety of disciplines in addition to healthcare providers, military leaders, and other stakeholders. Such language ought to be built on consensus and limit the use of discipline-specific jargon.

The purpose of the present paper is to begin a scientific dialogue regarding terminology related to blast exposure. To accomplish this, we first summarize descriptive conventions regarding the language scientists currently use when describing blast-related exposures and outcomes in both written and oral formats. This summary is based on findings from an extensive scoping review of the blast exposure research published within the past two decades (5). Second, we suggest prescriptive conventions about how these terms should be used by clearly conceptualizing relevant constructs (including elaborating on relevant distinctions that are under-articulated in the existing scientific literature) and providing explicit definitions for each term. After reviewing the distinction between conceptual and operational definitions, we move to a summary of terms that have been used in published literature to date to refer to HLB and LLB, respectively. We next summarize some essential concepts relevant to the study of blast for the purpose of conceptualizing and labeling relevant phenomena. We then provide a clear conceptualization of the distinction between HLB and LLB. Lastly, we close with a discussion of the use of the modifiers “acute” and “chronic” with regard to specific exposures and outcomes relevant to the study of blast exposure.

## Operational vs. Conceptual Definitions

Before discussing terminology related to blast exposure, it is important to clarify the crucial distinction between conceptual and operational definitions. Any given scientific concept should be described and defined both conceptually and operationally. Conceptual definitions are abstract summaries of the meaning of terms that can be applied across a range of scientific disciplines, study designs, and methodologies. Operational definitions, on the other hand, are concrete summaries of how a given conceptual definition is applied within a specific study. For example, a conceptual definition of blast exposure may refer to being in close enough proximity to feel a change in pressure following detonation of an explosive device, while a corresponding operational definition may specify the specific nuances of such exposure (e.g., the source, distance from the source, intensity, frequency, and duration of the exposure).

For the purpose of this paper, we focus on clarifying conceptual definitions of terminology related to the scientific study of the health effects of blast exposure in a way that is accessible to researchers from different disciplines with the goal of working toward unification and consensus. Due to the diverse methods used by subject matter experts to examine blast exposure, it is far beyond the scope of this paper to discuss operational definitions except when appropriate to provide accessible examples. Instead, we refer readers to findings presented in Belding et al. (5), which describe the operational definitions used in the study of LLB over the past two decades.

## Descriptive Conventions

### Expansion of a Previous Scoping Review

In a recent effort to summarize the extant scientific literature on LLB, we conducted a scoping review of peer-reviewed papers on the health-related effects of blast overpressure published between 2000 and 2019 (5). In the process of conducting this review, we recognized that a lack of standardization and consistency in terminology used by scientists to describe both HLB and LLB hindered our ability to complete a thorough search, and thus our ability to accurately characterize and categorize the literature. As a result, we undertook extensive steps to ensure the comprehensiveness of our search using a variety of search terms including blast injury(ies), blast exposure(s), blast wave(s), as well as the co-occurrence of the term blast with each of the following terms: bullets, wounds, low-level, low pressure, low intensity, lung, force, trauma, traumatic, concussion, induced, pressure, overpressure, and over pressure (e.g., “blast and bullets,” “blast and wounds”). This search resulted in nearly 5,600 articles for potential inclusion. The title and abstract of each article were reviewed to determine if they were relevant to HLB and/or LLB, which identified a total of 3,215 articles for inclusion. Of these, 51 articles were identified as LLB-relevant (see Appendix A), which included 23 empirical articles involving animal subjects, 20 empirical articles involving human participants, 1 article involving computational modeling, and 7 non-empirical articles (including literature reviews or commentaries). Because the focus of this prior review was on the health-related effects of LLB, the full text of each of these 51 articles was reviewed, and relevant information pertaining to study design and limitations were extracted (see reference 5 for a full explanation of methods).

During the full text review, we also extracted and categorized all terminology used to refer to blast exposure into groups corresponding to what we present in this paper as HLB and LLB, respectively. If explicit definitions for any term were provided within the article, we extracted those definitions. In order to identify the most prevalent terms in the literature, we defined commonly used terms those which appear in ~10% or more of peer-reviewed published articles on the topic, which corresponds to being used in at least five articles. In this article, we present a brief summary of this effort in order to articulate the descriptive conventions used to refer to blast overpressure in the peer-reviewed published literature to date.

### Results of Review

#### HLB-Related Terminology

A total of 157 terms were used to describe HLB. Only 11 of these terms (7%) met our threshold of being “commonly used” (see Table 1 for the commonly used terms and the Appendix B for the full list). Explicit definitions for HLB-related terminology were rare. For example, the term “blast exposure” was not defined in any article. The four terms describing HLB that were defined in the literature were blast overpressure, moderate-to-high intensity blast, operational blast exposure, and overpressure (Table 2). Additionally, terms referring to different types of blast-induced injury (i.e., primary, secondary, tertiary, quaternary, and quinary blast injury) were occasionally defined, likely due to clear articulation of definitions in existing military policy (Table 3).

**Table 1:** Commonly used terms to describe high-level blast (HLB) and low-level blast (LLB), respectively.

**HLB terminology**		**LLB terminology**	
**Term**	***N* (%)**	**Term**	***N* (%)**
Blasts	28 (55)	Low-level blasts	15 (29)
Blast exposure	19 (37)	Low-level blast exposure	8 (16)
Blast waves	17 (33)	Blasts	7 (14)
Blast overpressure	15 (29)	Primary blast	7 (14)
Primary blast injury	8 (16)	Primary blast injuries	6 (12)
Explosive blasts	7 (14)	Blast overpressure	5 (10)
Improvised explosive devices	7 (14)	Repeated blast exposure	5 (10)
Primary blast wave	6 (12)	Repeated low-level blasts	5 (10)
Secondary blast injury	5 (10)		
Tertiary blast injury	5 (10)		
Quaternary blast injury	5 (10)		

*To be included in this list, terms must have been used in at least 5 of the 51 articles included in the scoping review (see Appendix A)*.

**Table 2:** Explicit definitions provided in peer-reviewed published literature for high-level blast (HLB) and low-level blast (LLB) exposure, respectively.

**Term**	**Definition**
**HLB terminology**	
Blast overpressure	“Blast overpressure is largely accepted as an important traumatic mechanism given that up to 90% of the energies released on detonation of an uncased charge are converted into the formation of the shock wave” (8) (p. 30) “Blast overpressure (BOP) refers to a high-intensity disturbance in ambient air pressure” (9) (p. 33) “Blast overpressure (BOP), also known as high energy impulse noise, is a damaging outcome of explosive detonations and firing of weapons. Exposure to BOP shock waves alone results in injury predominantly to the hollow organ systems such as auditory, respiratory, and gastrointestinal systems” (10) (p. 289)
Moderate-to-high intensity blast	“…> 100 kPa peak overpressure…” (11) (p. 1,591)
Operational blast exposure	“Operational blast exposure, such as that from improvised explosive devices, exposes service personnel to multiple mechanisms of injury, including primary overpressure exposure, secondary penetrating fragmentation injury, tertiary blunt force trauma, and quaternary “miscellaneous” injury” (12) (p. 1,621).
Overpressure	“Proximity to a blast explosion results in exposure to an overpressure wave and can result in injury to the brain and body. In the military, overpressures occur due to a variety of sources including artillery and improvised explosive devices.” (13) (p. 1).
**LLB terminology**	
Chronic low-level overpressure	“Based on studies using the WRAIR shock tube, a dividing line seems to exist between 74.5 and 116.7 kPa that separates low-level blast in rats from moderate to higher level blast exposures that are more equivalent pathologically to human moderate to severe TBI in the context of polytrauma” (9) (p. 6)^a^
Subclinical blast	“…blast with no obvious sign of external trauma or lung injury…” (14) (p. 150)
Subclinical blast exposure	“…from door charges, concussive grenades, large-caliber-weapon muzzle overpressure, mortar training, antitank weaponry, artillery, and combatives training.” (15) (p. 55)
Explosive breaching	“These exposures are lower in explosive yield than exposures encountered with uncontrolled enemy weapons such as IEDs. They are, nonetheless, blast events with overpressures that have been measured to be well-beyond the safety standard of 4 psi” (16) (p. 48)
Career blast exposures	“…activities such as combat breaching and shoulder-fired heavy weapons…” (17) (p. 850)
Incident overpressure exposure	“Incident overpressure is often described as the pressure collected parallel to a blast wave streamline” (18) (p. 838)
Reflected pressure	“Reflected pressure is defined as the sum of static, dynamic, and reflective wave pressure components and can be thought of as the maximum or total pressure that could be read with a given pressure sensing element oriented orthogonal to (facing) a single streamline” (18) (p. 838)
Primary blast injuries	“…refers to the barotrauma from the overpressure effects of the explosion.” (19) (p. S472) “…injuries are due to the direct effects of the blast wave…” (20) (p. 2)
Tertiary blast injury	“…injuries involves displacement of the entire body and impact with other objects…” (20) (p. 2)
Quaternary injuries	“…refers to the other effects such as heat, chemical, or electromagnetic wave generation.” (20) (p.2)

a *The authors followed this definition with a discussion on how this may not apply beyond rats.*

*This table does not include definitions of terms referring to blast-induced injury (e.g., primary blast injury) because such terms were often defined in accordance with existing military policy*.

**Table 3:** Blast-induced injury terminology and definitions as provided in Department of Defense Directive “Medical Research for Prevention, Mitigation, and Treatment of Blast Injuries” (DoDD 6025.21E, July 5, 2006).

**Term**	**Definition**
Primary blast injury	“Blast overpressure injury resulting in direct tissue damage from the shock wave coupling into the body.”
Secondary blast injury	“Injury produced by primary fragments originating from the exploding device (performed and natural (unformed) casing fragments, and other projectiles deliberately introduced into the device to enhance the fragment threat); and secondary fragments, which are projectiles from the environment (debris, vehicular metal, etc.).”
Tertiary blast injury	“Displacement of the body or part of the body by the blast overpressure causing acceleration/deceleration to the body or its parts, which may subsequently strike hard objects causing typical blunt injury (translational injury), avulsion (separation) of limbs, stripping of soft tissues, skin speckling with explosive product residue and building structural collapse with crush and blunt injuries, and crush syndrome development.”
Quaternary blast injury	“Other “explosive products” effects—heat (radiant and convective) and toxic, toxidromes from fuel, metals, etc.—causing burn and inhalation injury.”
Quinary blast injury	“Clinical consequences of “post detonation environmental contaminants” including bacteria (deliberate and commensal, with or without sepsis), radiation (dirty bombs), tissue reactions to fuel, metals, etc.”

#### LLB-Related Terminology

A total of 226 terms were used to describe LLB. Only eight terms (4%) met our threshold of being “commonly used” (Table 1). Although the term “low-level blast” was the most frequently used term, it was still used in fewer than 30% of articles. Explicit definitions for various LLB-related terms were also rare, with only 10 terms explicitly defined in the literature (Table 2).

#### Duplicated Terminology

A close comparison of the lists of terms describing HLB and LLB revealed 16 terms that have been used to describe both types of blast in different, and sometimes even the same, published articles. These terms included acute to subacute effects of blast, blast exposure(s), blast overpressure injury, blast wave(s), blast(s), blasting, blast-related post-concussion syndrome, chronic blast exposure, impulse noise, isolated blast, overpressure exposure, primary blast, primary blast exposure, primary blast injury(ies), repeated blast overpressure, and tertiary blast (see Appendix B). Use of the same terminology to refer to different constructs can make it challenging for readers to correctly infer the author's intent, particularly across disciplines. Although context may help to clarify the authors' intent with such terms, correct understanding of these terms, even in context, presumes that readers have sufficient subject matter expertise and appropriate schemas to understand precisely what is meant. Thus, there is a need to clearly define and distinguish the terms related to blast exposure in general, which we address by offering the following prescriptive conventions.

## Prescriptive Conventions

Taken together, our scoping review identified a plethora of terms that have been used to describe the nature of blast exposures and corresponding outcomes yet revealed little consensus or consistency in the use of these terms. To help remedy this gap, we propose a clear, concise list of terminology for future use alongside a conceptual definitions that are intended to be accessible to experts from a wide variety of disciplines (see Table 4 for a summary). These conceptual definitions should be supplemented with operational definitions appropriate to each unique study design. In the following sections we review terms related to characterizing the nature of blast exposure. We then present a thorough articulation of the distinction between HLB and LLB. Finally, we discuss a conceptual matrix that differentiates both acute and chronic modifiers for exposures and outcomes, respectively.

**Table 4:** Glossary.

	**Term**	**Recommended conceptual definition**
Exposure terminology	Blast	An event that produces a shockwave/soundwave, resulting in a rapid change in atmospheric pressure
	Overpressure	Any transient increase above atmospheric pressure
	Blast event	Specific blast exposure(s) that occur(s) within a specified chronological time frame with clearly distinguishable start and end points; a single blast event can include multiple blast exposures if these were sustained during a single, specific period of time
	Blast exposure	Blast exposure occurs when an individual is close enough to a blast (HLB or LLB) to experience (e.g., physically feel) the shockwave
	Occupational blast exposure	Overpressure blast exposures experienced in the course of performing one's job
	Low-level blast (LLB) exposure	Overpressure exposure generally occurring within operational and training environments from outgoing (user directed) munitions
	High-level blast (HLB) exposure	Overpressure exposure generally experienced in combat settings as a result of incoming or enemy-inflicted munitions, such as IEDs, rocket-propelled munitions, etc.
Overpressure characterization	Peak overpressure	The highest recorded change above ambient pressure (overpressure); typically measured in pounds per square inch (psi) or kilopascals (kPa)
	Impulse overpressure	The total amount of change in pressure over time resulting from a single source; typically measured in pounds per square inch per millisecond (psi/ms)
	Reflective overpressure	The phenomenon in which pressure wave(s) hit object(s), combine, and magnify overpressure
Measurement terminology	Incident sensor orientation	Method of measuring change in ambient static pressure at 90 degrees to the blast origin
	Reflective Sensor Orientation	Method of measuring change in ambient pressure, which includes both static and dynamic pressure, at any orientation other than 90 degrees to the blast origin
Adjectives frequently used to clarify exposures	Repeated	Modifies: exposures An objective term referring to a quantity of exposures >1
	Frequent	Modifies: events A subjective term referring to multiple exposures within a specified time frame, usually short duration
	Acute	Modifies: exposures Refers to an exposure resulting from a single origin at a specific point in time that is relatively short in duration and transient
	Chronic	Modifies: exposures Refers to multiple acute exposures sustained over a prolonged period of time
	Career	Modifies: exposures, events Refers to the sum of exposures/events that occurred over the duration of occupation (typically military career)
	Lifetime	Modifies: exposures, events Refers to the sum of exposures/events that occurred over the person's life span and can include a combination of exposures from multiple careers (e.g., military service and subsequent law enforcement careers) as well as any extra-career exposures
Adjectives frequently used to characterize outcomes	Acute	Modifies: outcomes Refers to outcomes that occur in close temporal proximity to the exposure and are short in duration
	Chronic	Modifies: outcomes Refers to outcomes that occur over time, not necessarily within close temporal proximity to the exposure

### What Is a Blast?

To understand what a blast is and how it can be characterized for the purpose of scientific study, we must first understand some basic blast physics. Pressure is the amount of force exerted over an area. For example, the weight of the Earth's atmosphere pressing against the Earth's surface is atmospheric pressure, which can differ based on where one is located (e.g., it is lower at the top of a mountain compared with sea level). The pressure exerted on an object within its immediate surroundings is called ambient pressure and can be affected by many factors (e.g., altitude or elevation, temperature, humidity). Deviations in pressure are operationally measured as changes from ambient pressure (e.g., a 4-unit increase in pressure assessed using pounds per square inch [psi] would result in a measurement of 4 psi). When such deviations are positive, we refer to it as overpressure; when the deviations are negative, we refer to it as underpressure or negative pressure.

**Figure 1:**
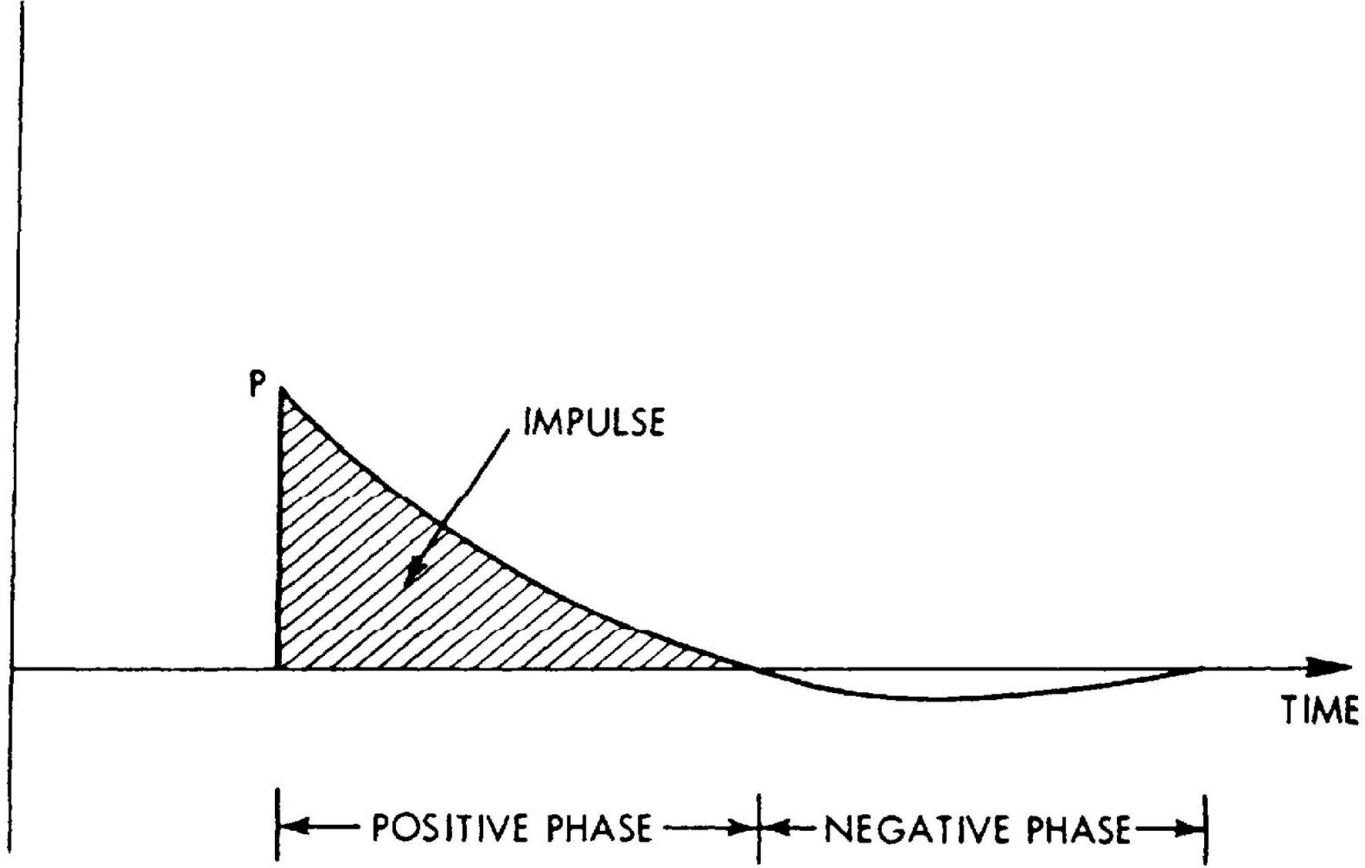
Graphical depiction of a Friedlander Curve (21).

A blast is one factor that can change ambient pressure. By definition, a blast is a rapid change in pressure (often from an explosion) that emits a shockwave, or highly compressed air wave, that travels outward radially from the blast origin at supersonic volumes. This shockwave temporarily affects ambient pressure. Because shockwaves dissipate over time and distance, the changes in atmospheric pressure can be modeled using a Friedlander curve, which demonstrates how the change in pressure (noted on the y-axis) changes over time (noted on the x-axis; see Figure 1). These shockwaves are characterized by a rapid rise in pressure above ambient pressure (i.e., positive y-values, because y = 0 indicates no change in ambient pressure), which decreases over time (including into a brief period where the curve goes below the x-axis, indicating negative pressure), and finally stabilizes back at baseline. The highest point recorded on the Friedlander curve is referred to as peak overpressure, while impulse pressure is indicated by the area under the curve and represents the total force imparted on an object over time. If one visualizes a shockwave as a tsunami, peak overpressure refers to the height of the wave at its highest point, whereas impulse pressure refers to the total volume of water that goes over land.

The strength or intensity of a given shockwave is correlated with distance from the source of the blast (along with a variety of other factors) and can be assessed with appropriate equipment, which must be used properly to obtain measurements that are both reliable and valid. For example, because shockwaves can bounce off nearby objects, features of the environment may alter the change in pressure compared with atmospheric pressure. This phenomenon is known as reflective overpressure. The sensor's orientation to the source of the blast may influence the sensor's ability to provide a reliable and valid output of the strength or intensity of the blast. When the sensor is placed with a perfect 90-degree orientation to the source of the blast (i.e., incident sensor orientation), it measures static overpressure and provides the most stable measure of overpressure across different environments. When the sensor is placed using anything other than a perfect 90-degree orientation (i.e., reflective sensor orientation), the reading generated may include both static and dynamic changes in pressure, some of which may result from reflection off of the sensor mount surface. These readings are subject to the environment in which the blast occurs and therefore can complicate comparisons across blast environments. For further information on these complexities, we refer readers to (22). One way of thinking about this is that incident sensor orientation is able to more accurately capture the true “signal” of the primary blast wave, whereas reflective sensor orientation captures this “signal” as well as potential “noise.” However, this analogy is somewhat limited because the “noise” captured can be meaningful as it may include both static and dynamic pressure as well as other elements of the complex environment in which a blast occurs. These measurement considerations may have important implications for conclusions garnered from a given study, and ultimately our understanding of the health-related consequences of blast exposure.

### Characterizing Blast Exposure

With the above description of what a blast is and how it is measured, we define blast exposure as that which occurs when an individual is close enough to a blast to be physically subjected to the shockwave. In essence, someone is exposed to blast if they are close enough to the blast to physically feel the shockwave (assuming it does not render them unconscious), just as a concertgoer would feel the soundwaves from speakers physically pound their chest if standing close enough to the stage. Blast events can include either single or multiple exposures from one or more sources (e.g., a series of door breaching charges), provided that these exposures are all sustained within a specified time frame with clearly distinguishable beginning and end points.

Because no two blasts are identical and blasts often occur in immensely chaotic real-world environments (e.g., in combat, during terrorist attacks), researchers must adequately characterize the nature of the blast event(s) to which the subject was exposed in order to understand the implications of blast exposure for health. For example, blast exposure can result from a variety of munitions (e.g., rocket-propelled grenades, IEDs, explosive charges), each with different characteristics. Presenting only a single value indicating overpressure exposure (e.g., “12 psi”) is insufficient because it is unclear whether the value refers to incident or reflective sensor orientation. To effectively characterize the blast exposure in a given study, researchers ought to report the source, frequency, duration, peak, and impulse overpressure (with clarification on whether it was assessed using incident or reflective sensor orientation), environment (e.g., open field), and whether personal protective equipment was used. It is incumbent upon scientists to provide as much of this information as possible and to do so using language that is as clear and specific as possible, as we have laid out here.

### Distinguishing HLB From LLB

#### Current Convention

Previous research on the health-related consequences of HLB and LLB has demonstrated the potential for harm (23, 24), yet a clear articulation of the distinction between HLB and LLB has not been provided in the scientific literature to date. The most common current convention to distinguish HLB from LLB is based on a 4 psi peak overpressure threshold (measured using incident sensor orientation), with values above and below 4 psi corresponding to HLB and LLB, respectively (25). The use of 4 psi as a threshold emerged from research that suggests that 4 psi is the cutoff for safe exposure with respect to rupture of the tympanic membrane, also referred to as the eardrum, an inner structure of the ear necessary for hearing (26). As such, the 4 psi distinction is utilized in calculating minimum safe standoff distances in military operations and training (e.g., breacher training).

Although using a 4 psi shift from ambient pressure as the threshold for differentiating HLB and LLB has the advantage of being easily operationalized, the broad acceptance of this method of differentiation is problematic. While this threshold may be used to effectively identify minimum safe standoff distances for tympanic membrane damage (27), its utility regarding assessments of safe distances to protect other body regions has not been established. Given remarkable variability in the structure and function of different regions of the body, it is not only plausible but likely that the level of overpressure that can be sustained without damage will vary across body regions. This point has not received adequate attention. Furthermore, there is no scientific consensus to date suggesting that a 4 psi peak overpressure threshold is a cutoff for safe exposure to the brain, nor does it account for impulse pressure. Additionally, an intact tympanic membrane does not accurately predict whether a person may have other primary blast injuries such as damage to the brain or lungs (28, 29). It is also worth noting that the intensity of the overpressure to which a warfighter may be exposed differs based on proximity to the source of the blast, which complicates the articulation of the distinction between HLB and LLB when that distinction is based primarily on the intensity of overpressure experienced.

#### A New Delineation

Although a 4 psi peak overpressure threshold is *one* important element of the distinction between HLB and LLB, we believe that reliance on it as *the distinguishing factor* is misguided and could be hampering advances in understanding the consequences of blast for service member health and readiness. Instead, we argue that HLB and LLB differ on several critical dimensions including the source, setting, intensity, and predictability (see Table 5), *with the critical distinguishing factor being whether the source of the blast is from incoming or outgoing munitions*^1^. We define HLB as overpressure exposure that results from incoming enemy munitions (e.g., IEDs, rocket-propelled grenades). Detonation from such munitions tends to be unpredictable because if their location were to be known, additional measures would be taken to neutralize the threat. HLB is typically higher in intensity than LLB (often exceeding 4 psi peak overpressure using incident sensor orientation) and is generally experienced in combat settings; HLB exposure is unlikely to occur during routine training aside from accidents or mishaps. In contrast, we define LLB as overpressure exposure that results from outgoing (i.e., user directed) munitions being fired at an enemy or target. LLB is typically lower in intensity than HLB (often below 4 psi incident peak overpressure) and generally occurs in both operational and training environments. Such exposures are presumably more predictable than HLB exposures, assuming that the unit's operational tempo and mission are known. The additional benefit of our definitions of HLB and LLB is that they allow for differentiation that can on some level, help account for variability in impulse. For example, LLB from small arms fire can generate several psi of peak overpressure, but have extremely short impulses. The lower accumulation of total pressure likely attenuates the impact on health and wellness.

**Table 5:** Distinctions between high-level blast (HLB) and low-level blast (LLB).

	**HLB**	**LLB**
Examples	Improvised explosive devices (IEDs)	Carl Gustav bazooka, Howitzer cannon, explosive breaching charges
Source	Typically the result of incoming munitions; being on the receiving end of enemy-initiated weapon fire	Typically the result of outgoing munitions; being the source of the fired weapon
Setting	Typically occurs during operational settings and environments	Typically occurs during both training and operational settings and environments
Intensity	Typically higher in experienced pounds per square inch (psi)	Typically lower in experienced pounds per square inch (psi)
Predictability	Typically not predictable	Can presumably be predicted if one knows a unit's given operational tempo and training schedule
Occupation	Typically experienced by military personnel and shows heterogeneity within an individual occupation	Typically experienced by military and law enforcement personnel and shows homogeneity within an individual occupation
Corresponding physical injuries	Frequently associated with injuries beyond primary blast injury including secondary, tertiary, quaternary, and quinary blast injury	May be associated with primary blast injury, but is rarely associated with injuries beyond primary blast injury
Corresponding psychological stressors	Can involve psychological trauma, such as fearing for one's own life or seeing dead or maimed bodies, but is unlikely to be associated with guilt over harming others	Can involve psychological trauma, such as guilt over harming others, but is unlikely to be associated with fear for one's own life

If one accepts this multifaceted delineation of the differences between HLB and LLB, it is important to note that HLB and LLB are not two ends of a single continuum, but rather two distinct concepts that share some characteristics in common. In the real world, and particularly in the combat environment, it is likely that service members who are exposed to HLB (i.e., from enemy fire) are also simultaneously exposed to LLB from firing a weapon themselves. However, being exposed to LLB does not necessarily mean that a service member has also been subject to HLB. Furthermore, because firing on the enemy can occur during military operations, in addition to in training environments, we recommend against the term “operational blast exposure” unless one intends to simultaneously refer to both forms of blast exposure sustained during military operations because it does not clarify whether the exposure was due to HLB or LLB.

#### HLB, LLB, and Military Occupation

Given the more predictable nature of LLB exposure, we argue that there are occupational differences in the extent to which service members may be exposed to repetitive LLB. For example, in other work, we have argued that some military occupational specialties (e.g., Infantry, Artillery) are likely at greater risk of repetitive LLB exposure than others (e.g., Personnel and Administration) (30, 31). We further propose that there is more homogeneity within individual military occupations with regard to LLB exposure than HLB exposure. For example, when controlling for additional relevant characteristics (e.g., time in service), Artillerymen are likely to be exposed to relatively similar amounts of LLB, which generally exceeds the amount of LLB exposure to which those in Personnel and Administration occupations might be subjected. However, we do not believe this necessarily holds true for HLB. Instead, we argue that, all else being equal, HLB shows more heterogeneity across military occupations due to the unpredictable nature of combat environments.

#### HLB, LLB, and Health Outcomes

We further suggest that these two forms of overpressure may have different consequences for health and well-being. Both HLB and LLB have the potential to harm service members, but in different ways. Based on official military policy, physical blast injuries can be categorized as primary, secondary, tertiary, quaternary, and quinary (see Table 3). Although HLB can be associated with each of these forms of injury, current evidence suggests that LLB results at most in primary blast injury; there is currently no evidence in the peer-reviewed literature to suggest that LLB results in secondary, tertiary, quaternary, or quinary blast injury (32). For example, we would not expect an LLB event to be associated with shrapnel or physical displacement of one's body, as might be the case for an HLB event. Furthermore, ongoing research is examining the associations among HLB, LLB, and traumatic brain injuries (TBIs), which are typically categorized as mild (also known as concussion), moderate, severe, or penetrating (33). Although it is beyond the scope of this article to fully articulate findings to date on these associations, we note that HLB is associated with TBIs of all severity, whereas LLB may increase susceptibility to concussion but is currently believed to be unlikely to result in concussion in the absence of some other potentially TBI-inducing event (e.g., HLB, motor vehicle crash) (5, 24, 30, 31). Additionally, we conjecture that HLB and LLB may be associated with different psychological stressors (e.g., feeling responsible for another's death, seeing maimed, or wounded colleagues), though the nature of these stressors and the corresponding association with adverse mental health outcomes is poorly understood at this time and warrants future research.

### Acute and Chronic Exposures and Outcomes

Another set of expressions that are frequently misunderstood and misrepresented in the literature are the terms acute, chronic, exposures, and outcomes. The four terms correspond to two distinct, orthogonal concepts that can be represented by a 2 × 2 matrix. The terms acute and chronic refer to different ends of a continuum, while the terms exposures and outcomes are dichotomous. Unfortunately, these terms are often imprecise in the literature and usage may differ in publications summarizing preclinical vs. clinical research. It is not uncommon, for example, for discussions of the study of LLB to be framed as a study of chronic LLB. This lack of clarity may lead readers, for example, to incorrectly infer that both the exposure and outcomes are chronic when the study actually examines chronic exposures and acute outcomes. Furthermore, some terms (e.g., “subclinical blast exposure”) that are frequently used to refer to LLB conflate the exposure with the outcome; stated differently, researchers using such terminology are defining the presence of an exposure based on whether or not an outcome occurred, rather than separately considering the exposure, the outcome, and their association.

This ambiguity of what is acute vs. chronic and what is an exposure vs. an outcome is inherently problematic because it hinders the pursuit of research into moderators of associations between exposures and outcomes (e.g., factors that influence when or for whom HLB exposure will produce a loss or alteration of consciousness and thus TBI). Yet, a search for such moderators would be tremendously useful from a military perspective. This imprecise terminology also limits our ability to fully understand the scope of the consequences of various exposures because we will have arbitrarily excluded some outcomes from consideration (e.g., exposures that do not result in clinically diagnosable injury). The absence of an acute outcome does not mean that an exposure is “safe,” nor does the presence of an “exposure” mean that someone has been “harmed,” yet the ambiguous use of these terms in this way implies as much. We wish to make clear that there are really four combinations of these two concepts in that there can be acute exposures, chronic exposures, acute outcomes, and chronic outcomes. We next review specific examples of each of the four combinations of these terms.

#### Acute and Chronic Exposures

The distinction between acute and chronic is inherently temporal. Conceptually speaking, an acute exposure is an exposure that has a clearly delineated origin and end point that occurs at a specific point in time; it should be construed as an exposure that is transient and relatively short in duration. The term chronic exposure, in contrast, refers to multiple acute exposures sustained over a prolonged period of time. Articulation of the precise amount of time needed to distinguish between acute and chronic exposures is a question of operational definitions that ought to be specified for each study. According to these definitions, exposure to a single IED during deployment or a series of breaches during a day of operations would qualify as acute exposures since both have clearly delineated origins and end points. While it is possible that a service member could be exposed to multiple IEDs in different deployments (or even the same deployment), these should be construed as separate acute exposures because they have distinct beginning and end points.

In the case of blast exposure, HLB exposure is often acute, while LLB exposure is often considered chronic. However, there could be exceptions to this. For example, HLB exposure could be considered chronic if a service member were repeatedly exposed to HLB throughout a prolonged deployment or a series of deployments, depending on the researcher's operational definitions. Similarly, LLB exposure could be considered acute if a service member assisted with firing a specific weapon system after the weapon's normal operator was injured or killed in combat. Although we believe that HLB exposures are generally distinct acute exposures and LLB exposure is generally chronic, for clarity we highly encourage researchers to provide operational definitions for what makes something acute vs. chronic within a particular setting or environment.

#### Acute and Chronic Outcomes

Regardless of whether the exposure is acute or chronic, each type of exposure can have acute and/or chronic outcomes. Acute outcomes are defined as outcomes that occur in close temporal proximity to the exposure and are short in duration. Chronic outcomes, in contrast, are those that continue to occur over time (34). Providing further clarification for what constitutes a “short duration” (i.e., acute) vs. a “long duration” (i.e., chronic) crosses the boundary between conceptual definitions and operational definitions and again must be determined for each outcome of interest based on existing understanding. In the case of medical outcomes, such delineations are often provided by diagnostic criteria. For example, imagine a service member who experiences headaches following a concussion. According to current diagnostic criteria, if these headaches resolve within 3 months, they are considered acute. If these headaches persist for 3 months or more, they are considered persistent (chronic) and would prompt a new diagnosis of post-concussive syndrome (19).

Expanding on our earlier distinction between HLB and LLB, we urge scientists to take care not to conflate the exposure with the outcome by using the outcome to define the exposure. That is, LLB exposure should be defined independent of whether it produces any adverse outcome. Although scientists have used the phrase “subclinical blast exposure” to refer to LLB, this term inherently conflates the experience of the exposure with an outcome (i.e., subclinical levels of harm). Because the term subclinical refers to an outcome that has not reached the level of clinically diagnosable injury, the phrase “subclinical blast exposure” would thus only define the exposure based on whether or not it resulted in adverse outcomes. These distinctions also raise the question of what rises to the level of a clinical diagnosis, which is an important issue but beyond the scope of this paper.

We suggest that the concept of injury inherently occurs along a continuum from non-injured to severely injured. Although medical diagnoses often serve as a cut point on this continuum and allow people to be labeled as “injured,” we note that the absence of a clinically diagnosable injury, whether acute or chronic, does not necessarily mean that someone is well. In this sense, even “subclinical” injuries can be harmful, particularly if one considers the full range of outcomes (such as delayed reaction time, irritability, trouble with decision making) associated with blast exposures (12, 35). Although “subclinical” outcomes would not qualify as clinical diagnoses of injury, these sequelae of blast exposure are still meaningful to understand from a scientific perspective (36). It should also be noted that the term “subclinical blast exposure” is also problematic for another reason: Although the intent is to use the term to describe LLB, it could also technically be appropriately used to describe an acute HLB exposure from which a service member was far enough away so as not to sustain a clinically diagnosable injury.

## Discussion

Although research on blast injury is progressing at a remarkable rate due to dedicated efforts from subject matter experts across a wide variety of disciplines, confusion, and lack of consistency regarding conceptualization of key concepts and articulation of appropriate terminology to describe these concepts remain common. In a scoping review of research on the potentially adverse effects of LLB exposure (5), we found that there are literally hundreds of terms used to describe blast exposure and very little consensus on the meaning of those terms. To address these issues, we have presented a clear, concise list of terminology and corresponding explicit definitions. Specifically, we (1) provided accessible definitions for key terms used to describe the nature of blast exposure, (2) clearly articulated a multifactorial distinction between HLB and LLB, and (3) clarified how the terms acute, chronic, exposure, and outcome ought to be used when referring to health-related consequences of blast exposure.

We do not presume that the articulation we have offered in this paper is perfect; rather, we hope that by presenting both descriptive and prescriptive conventions for conceptualizing and defining terminology for researchers studying the health effects of blast, we will generate increasing dialogue on these issues that will facilitate cross-disciplinary collaboration and help propel the field forward. We believe that achieving scientific consensus on relevant terminology can help clarify the many elements of blast exposure that ought to be studied in future research. By demystifying relevant jargon, the multidisciplinary scientific investigation of the health-relevant consequences of blast exposure can yield important insights for prevention, screening, and treatment of blast-induced injuries, which is essential to ensuring the health of members of the U.S. Armed Forces and civilians alike.

## Author Contributions

JB led the research effort. RE and ME contributed to the design of the effort. SF and JB extracted relevant data. JB analyzed the data. JB and ME equally contributed to the first draft of the manuscript. All authors contributed to the theoretical framework of the paper, interpretation of results, contributed to the development of this manuscript, provided comments, and revisions.

## Conflict of Interest

JB, RE, and SF were employed by company Leidos. The remaining authors declare that the research was conducted in the absence of any commercial or financial relationships that could be construed as a potential conflict of interest.

## Footnotes

^1^This articulation of HLB and LLB does not apply to accidents or mishaps in training.
