# Supplementary material for: Getting on the Same Page: Consolidating Terminology to Facilitate Cross-Disciplinary Health-Related Blast Research
Source: Front Neurol. 2021 Jun 24;12:695496. doi: 10.3389/fneur.2021.695496 (PMC8264539; doi:10.3389/fneur.2021.695496)
Supplement: Supplementary file 1 [file Data_Sheet_1.docx]

**Appendix A**

Ahlers S, Vasserman-Stokes E, Shaughness M, Hall A, Shear D, Chavko M, et al. Assessment of the effects of acute and repeated exposure to blast overpressure in rodents: toward a greater understanding of blast and the potential ramification for injury in humans exposed to blast. *Front Neurol*. (2012) 3:32. doi: 10.3389/fneur.2012.00032

Ahmed, F, Plantman S, Cernak I, Agoston DV. The temporal patttern of changes in serum biomarker levels reveals complex and dynamically changing pathologies after exposure to a single low-intensity blast in mice. *Front Neurol*. (2015) 6:114. doi: 10.3389/fneur.2015.00114

Baker AJ, Topolovec-Vranic J, Michalak A, Pollmann-Mudryj M-A, Ouchterlony D, Cheung B, Tien HC. Controlled blast exposure during forced explosive entry training and mild traumatic brain injury. *J Trauma*. (2011) 71:S472–7. doi: 10.1097/TA.0b013e318232e7da

Blennow K, Jonsson M, Andreasen N, Rosengren L, Wallin A, Hellström PA, Zetterberg H. No neurochemical evidence of brain injury after blast overpressure by repeated explosions or firing heavy weapons. *Acta Neurol Scand*. (2011) 123:245–51.

Bonnette S, Diekfuss JA, Kiefer AW, Riley MA, Barber Foss KD, Thomas S, et al. A jugular vein compression collar prevents alterations of endogenous electrocortical dynamics following blast exposure during special weapons and tactical (SWAT) breacher training. *Exp Brain Res*. (2018) 236:2691–701. doi: 10.1007/s00221-018-5328-x

Capo-Aponte JE, Jurek GM, Walsh DV, Temme LA, Ahroon WA, Riggs DW. Effects of repetitive low-level blast exposure on visual system and ocular structures. *J Rehabil Res Dev*. (2015) 52:273–90.

Carr W, Dell KC, Yanagi MA, Hassan, DM, LoPresti ML. Perspectives on repeated low-level blast and the measurement of neurotrauma in humans as an occupational exposure risk. *Shock Waves*. (2017) 27:829–36.

Carr W, Polejaeva E, Grome A, Crandall B, LaValle C, Eonta SE, Young LA. Relation of repeated low-level blast exposure with symptomology similar to concussion. *J Head Trauma Rehabil*. (2015) 30:47–55.

Carr W, Stone JR, Walilko T, Young LA, Snook TL, Paggi ME, et al. Repeated low-level blast exposure: a descriptive human subjects study. *Mil Med*. (2016) 181:28–39.

Carr W, Yarnell AM, Ong R, Walilko T, Kamimori GH, da Silva U, et al. Ubiquitin carboxy-terminal hydrolase-L1 as a serum neurotrauma biomarker for exposure to occupational low-level blast. *Front Neurol*. (2015) 6:49. doi: 10.3389/fneur.2015.00049

Chen T, Smith K, Jiang S, Zhang T, Gan RZ. Progressive hearing damage after exposure to repeated low-intensity blasts in chinchillas. *Hearing Res*. (2019) 378:33–42.

Choi JH, Greene WA, Johnson AJ, Chavko M, Cleland JM, McCarron RM, Wang H-C. Pathophysiology of blast-induced ocular trauma in rats after repeated exposure to low-level blast overpressure. *Clin Exp Ophthalmol*. (2015) 43:239–46.

Conley YP, Diaz-Arrastia R. Genomic links between blast exposure, brain injury, and Alzheimer disease. *Neurol Genet*. (2017) 3:e196.

Dams-O’Connor K, Tsao JW. Functional decline 5 years after blast traumatic brain injury: sounding the alarm for a wave of disability? *JAMA Neurol*. (2017) 7:763–4.

Elder GA, Stone JR, Ahlers ST. Effects of low-level blaast exposure on the nervous system: is there really a controversy? *Front Neurol*. (2014) 5:269.

Elsayed NM, Gorbunov NV. Pulmonary biochemical and histological alterations after repeated low-level blast overpressure. *Toxicol Sci*. (2006) 95:289–96.

Gama Sosa MA, De Gasperi R, Perez Garcia GS, Perez GM, Searcy C, Vargas D, et al. Low-level blast exposure disrupts gliovascular and neurovascular connections and induces a chronic vascular pathology in rat brain. *Acta Neuropathol Commun*. (2019) 7:6.

Gama Sosa MA, De Gasperi R, Perez Garcia GS, Sosa H, Searcy C, Vargas D, et al. Lack of chronic neuroinflammation in the absence of focal hemorrhage in a rat model of low-energy blast-induced TBI. *Acta Neuropathol Commun*. (2017) 5:80.

Gill J, Cashion A, Osier N, Arcurio L, Motamedi V, Dell KC, et al. Moderate blast exposure alters gene expression and levels of amyloid precursor protein. *Neurol Genet*. (2017) 3:e186.

Gill J, Motamedi V, Osier N, Dell K, Arcurio L, Carr W, et al. Moderate blast exposure results in increased IL-6 and TNFα in peripheral blood. *Brain Behav Immun*. (2017) 65:90–4.

Hall AA, Mendoza MI, Zhou H, Shaughness M, McCarron RM, Ahlers ST. Repeated low intensity blast exposure is associated with damaged endothelial glycocalyx and downstream behavioral deficits. *Front Behav Neurosci*. (2017) 11:104. doi: 10.3389/fnbeh.2017.00104

Kamimori GH, LaValle CR, Eonta SE, Carr W, Tate C, Wang KKW. Longitudinal investigation of neurotrauma serum biomarkers, behavioral characterization, and brain imaging in soldiers following repeated low-level blast exposure (New Zealand Breacher Study). *Mil Med*. (2018) 183:28–33.

Kamimori GH, Reilly LA, LaValle CR, Olaghere Da Silva UB. Occupational overpressure exposure of breachers and military personnel. *Shock Waves*. (2017) 27:837–47.

Kubli LR, Pinto RL, Burrows HL, Littlefield PD, Brungart DS. The effects of repeated low-level blast exposure on hearing in marines. *Noise Health*. (2017) 19:227–38. doi: 10.4103/nah.NAH_58_16

Kulik A, Malinowska-Borowska J. Exposure to impulse noise at an explosives company: a case study. *Int J Occup Saf Ergon*. (2018) 24:366–72. doi: 10.1080/10803548.2017.1420583

Li Y, Yang Z, Liu B, Valdez C, Chavko M, Cancio LC. Low-level primary blast induces neuroinflammation and neurodegeneration in rats. *Mil Med*. (2019) 184:265–72.

Lien S, Dickman JD. Vestibular injury after low-intensity blast exposure. *Front Neurol*. (2018) 9:297. doi: 10.3389/fneur.2018.00297

Littlefield PD, Pinto RL, Burrows HL, Brungart DS. The vestibular effects. *J Neurotrauma*. (2016) 33:71–81.

McBride DI, Williams S. Air blast circuit breaker noise and hearing loss: a multifactorial model for risk assessment. *Occup Med*. (2000) 50:173–81. https://doi.org/10.1093/occmed/50.3.173

McBride DI, Williams S. Audiometric notch as a sign of noise induced hearing loss. *Occup Environ Med*. (2001) 58:46–51. doi: 10.1136/oem.58.1.46

Park E, Eisen R, Kinio A, Baker AJ. Electrophysiological white matter dysfunction and association with neurobehavioral deficits following low-level primary blast trauma. *Neurobiol Dis*. (2013) 52:150–9.

Perez-Garcia G, Gama Sosa MA, De Gasperi R, Lashof-Sullivan M, Maudlin-Jeronimo E, Stone JR, et al. Exposure to a predator scent induces chronic behavioral changes in rats previously exposed to low-level blast: implications for the relationship of blast-related TBI to PTSD. *Front Neurol*. (2016) 7:176. doi: 10.3389/fneur.2016.00176

Perez-Garcia G, Gama Sosa MA, De Gasperi R, Lashof-Sullivan M, Maudlin-Jeronimo E, Stone JR, et al. Chronic post-traumatic stress disorder-related traits in a rat model of low-level blast exposure. *Behav Brain Res*. (2018) 340:117–25.

Por ED, Choi J-H, Lund BJ. Low-level blast exposure increases transient receptor potential vanilloid 1 (TRPV1) expression in the rat cornea. *Curr Eye Res*. (2016) 41:1294–301.

Pun P, Kan EM, Salim A, Li ZH, Ng KC, Moochhala S, et al. Low level primary blast injury in rodent brain. *Front Neurol*. (2011) 2:19. doi: 10.3389/fneur.2011.00019

Rhea, CK, Kuznetsov NA, Ross SE, Long B, Jakiela JT, Bailie JM, et al. Development of a portable tool for screening neuromotor sequelae from repetitive low-level blast exposure. *Mil Med*. (2017) 182:147–54.

Saljo A, Arrhen F, Bolouri H, Mayorga M, Hamberger A. Neuropathology and pressure in the pig brain resulting from low-impulse noise exposure. *J Neurotrauma*. (2008) 25:1397–406.

Säljö A, Bao F, Hamberger A, Haglid KG, Hansson H-A. Exposure to short-lasting impulse noise causes microglial and astroglial cell activation in the adult rat brain. *Pathophysiology*. (2001) 8:105–11.

Saljo A, Bolouri H, Mayorga M, Svensson B, Hamberger A. Low-level blast raises intracranial pressure and impairs cognitive function in rats: propylaxis with processed cereal feed. *J Neurotrauma*. (2010) 27:383–9.

Säljö A, Mayorga M, Bolouri H, Svensson B, Hamberger A. Mechanisms and pathophysiology of the low-level blast brain injury in animal models. *Neuroimage*. (2011) 54:S83–8.

Song H, Chen M, Chen C, Cui J, Johnson CE, Cheng J, et al. Proteomic analysis and biochemical correlates of mitochondrial dysfunction after low-intensity primary blast exposure. *J Neurotrauma*. (2019) 36:1591–605.

Song H, Konan LM, Cui J, Johnson CE, Langenderfer M, Grant D, et al. Ultrastructural brain abnormalities and associated behavioral changes in mice after low-intensity blast exposure. *Behav Brain Res*. (2018) 347:148–57.

St Onge P, McIlwain DS, Hill ME, Walilko TJ, Bardolf LB. Marine Corps Breacher Training Study: auditory and vestibular findings. *US Army Med Dep J*. (2011) 97–107.

Suttles ST. Potential of visual sensory screening, diagnostic evaluation, and training for treatment of postconcussive symptoms and performance enhancement for Special Forces qualified personnel. *J Spec Oper Med*. (2015) 15:54–63.

Tate CM, Wang KKW, Eonta SE, Zhang Y, Carr W, Tortella FC, et al. Serum brain biomarker level, neurocognitive performance, and self-reported symptom changes in soldiers repeatedly exposed to low-level blast: a breacher pilot study. *J Neurotrauma*. (2013) 30:1620–30.

VandeVord PJ, Bolander R, Sajja VSSS, Hay K, Bir CA. Mild neurotrauma indicates a range-specific pressure response to low level shock wave exposure. *Ann Biomed Eng*. (2012) 40:227–36.

Weiner MW, Friedl KE, Pacifico A, Chapman JC, Jaffee MS, Little DM, et al. Military risk factors for Alzheimer’s disease. *Alzheimers Dement*. (2013) 9:445–51.

Wiri S, Ritter AC, Bailie, JM, Needham C, Duckworth JL. Computational modeling of blast exposure associated with recoilless weapons combat training. *Shock Waves*. (2017) 27:849–62.

Yuan W, Foss KDB, Dudley J, Thomas S, Galloway R, DiCesare C, et al. Impact of low-level blast exposure on brain function after a one-day tacticle training and the ameliorating effect of a jugular vein compression neck collar device. *J Neurotrauma*. (2019) 36:721–34.

Zuckerman A, Ram O, Ifergane G, Matar MA, Sagi R, Ostfeld I, et al. Controlled low-pressure blast-wave exposure causes distinct behavioral and morphological responses modelling mild traumatic brain injury, post-traumatic stress disorder, and comorbid mild traumatic brain injury–post-traumatic stress disorder. *J Neurotrauma*. (2017) 34:145–64.

**Appendix B**

*Additional terms related to high-level blast (HLB)*

Acute blast; Acute blast effect; Acute blast exposure; Acute Blast overpressure exposure; Acute BOP exposure; Acute to subacute effects of blast; Acute-high level blast exposure; Blast attacks; Blast entries; Blast environments; Blast event; Blast explosion; Blast explosive waves; Blast exposure from an improvised explosive device; Blast exposure(s); Blast forces; Blast in combat; Blast induced injury(ies); Blast injury(ies); Blast intensity levels; Blast levels; Blast loading; Blast magnitude; Blast overpressure (BOP); Blast overpressure exposure; Blast overpressure injury; Blast overpressure shock wave; Blast overpressures; Blast pathophysiology; Blast pressure exposure; Blast scenarios; Blast shock wave; Blast TBI; Blast test area; Blast wave exposure; Blast wave(s); Blast(s); Blast-associated sensory and cognitive trauma; Blast-exposure; Blast-induced neurological deficits; Blast-induced neurotrauma; Blast-induced ocular injury; Blast-induced TBI; Blast-induced TBI (biTBI); Blast-induced traumatic brain injury; Blasting; Blast-pressure wave exposure; Blast-related barotraumas; Blast-related head trauma; Blast-related mild traumatic brain injury; Blast-related mTBIs; Blast-related postconcussion syndrome; Blast-related sensory trauma; Blast-related TBI; Blasts at close range; Blast-wave overpressure peak; Blast-wave trauma; BOP shockwaves; BOP wave; C4 high explosive; Chronic blast exposure; Clinical blast exposure; Close range explosives; Combat blasts; Detonation pulse; Detonation(s); Expanding blast wave; Explosion(s); Explosive blast exposures; Explosive blast injury; Explosive blast(s); Explosive blast-wave exposure; Explosive detonations of conventional or improvised explosives; Explosive event; Explosive impulses; Explosive munitions; Explosive pressure wave; Exposure to blast; Exposure to improvised explosive devices and/or other battle field related blast waves; High explosive blasts; High explosive blasts during combat; High explosives; High intensity blast exposures; High intensity blasts; High levels of blast overpressure; High overpressure blast levels; High pressure blast waves; High-energy blast exposures; High-energy blast waves; High-energy impulse noise; High-energy open-field explosions; High-energy, high-velocity blast waves; Higher intensity blast exposure; Higher level blast exposure; Higher-level blast; High-explosive charges; High-impact blast; High-intensity blast; High-intensity blast exposures; High-intensity blast waves; High-intensity blasts; High-level blast exposure(s); High-level blasts; High-level BOP; High-pressure blast waves; IED blast; Improvised explosive devices (IEDs); Impulse noise; Impulse noise exposure; Injuries from secondary blast; Isolated blast; Isolated blast exposure; Large magnitude blast; Military blast exposure; Moderate blast cases; Moderate primary blast exposure; Moderate to high BOP exposure; Moderate- to high-intensity explosions; Moderate to severe blast injury; Moderate-to-high-intensity blast; Multiple blast exposure; Open file exposure; Open-field shockwave; Operational blast exposure; Overpressure exposure; Overpressure wave(s); Overpressure(s); Overpressurization wave; Pressure required to induce severe, blast-induced TBI; Pressure waves; Primary blast; Primary blast exposure; Primary blast injury(ies); Primary blast shock wave; Primary blast wave(s); Primary blast-induced brain injury; Primary explosion; Primary overpressure exposure; Propagating pressure wave; Quaternary Blast injury(ies); Quinary blast injury; Relatively high-energy blast; Relatively high-intensity blasts; Relatively powerful blast exposure(s); Repeated blast overpressure; Repeated blast overpressure on mission; Repeated exposure to blast overpressure; Secondary blast injury(s); Shock wave; Single blast exposure; Single blast overpressure; Stronger explosive blasts; Tertiary blast; Tertiary blast injury(ies); Traumatic exposure to explosive impulses; Underwater detonation blast overpressure; War-related blast injuries

*Additional terms related to low-level blast (LLB)*

30-kPa blast; 60-kPa blast; Acute to subacute effects of blast; Air blast; Air blast circuit breaker noise exposure; Air blast circuit breakers; Air blast exposure; Back blast; Blast as part of combat training; Blast as part of routine combat training with should-fire recoilless rifles; Blast breaching exposures; Blast environment; Blast exposure(s); Blast from firing of military weapons; Blast of high pressure air; Blast overpressure; Blast overpressure from these shoulder-fired weapons; Blast overpressure injury; Blast overpressure waves; Blast overpressures of 21-35kPa; Blast related mTBI; Blast training and repeated blast exposure; Blast wave(s); Blast(s); Blasted; Blasting; Blast-related mTBI; Blast-related postconcussion syndrome; Breach blast pressure; Breacher blast; Breaching; Breaching blast; Career blast exposure; Chronic blast exposure; Chronic effects of lower level blast exposure; Chronic exposure to low-level blasts; Chronic exposure to repetitive low-level blast; Chronic low-level blast; Chronic low-level overpressure; Combat training with explosive breaching; Continuous noise; Controlled blasts; Cumulative, repeat, low-level blast; Explosive blast training; Explosive breaching; Explosive training; Exposure to air blast circuit breaker noise; Exposure to blasts below levels that cause such a loss of consciousness is defined as subclinical or a mild TBI ; Exposure to occupational levels of low-level blast; Exposure to repeated low intensity blast; Exposure to repeated low-level blast; Exposures to low-level blast events; Firearm exposure; Gunfire exposure; Heavy weapon blasts; Impulse noise; Impulse noise encountered in the workplace; Impulse noise from air blast breakers; Incident overpressure exposure; Isolated blast; Less intense forms of blast exposure; LLB overpressure; Long-term low-level blast exposure; Low blast exposure; Low BOP intensities; Low intensity blast exposure; Low intensity blast-induced brain injury; Low intensity shock wave; Low level primary blast; Low levels of blast; Low levels of blast exposure; Low to moderate blast levels; Low, occupational levels of blast exposure; Low-energy blast exposures; Lower blast pressures; Lower level exposures; Lower level exposures associated with mild traumatic brain injury (mTBI); Lower level exposures producing mTBIs; Lower-level blast exposures; Lower-level energy blast; Lower-level energy blast exposures; Low-intensity blast; Low-intensity blast (LIB); Low-intensity blast exposure(s); Low-intensity blast overpressure; Low-intensity blast wave exposure; Low-intensity explosive blast; Low-intensity explosive blast-waves; Low-intensity primary blast injury; Low-intensity primary blast waves; Low-level blast (LLB) exposure; Low-level blast events; Low-level blast exposure to BOP; Low-level blast exposure(s); Low-level blast injury; Low-level blast overpressure(s); Low-level blast pressure waves; Low-level blast(s); Low-level BOP; Low-level explosive blast; Low-level explosive charge; Low-level exposure(s); Low-level primary blast; Low-level primary blast exposure; Low-level repeated blast exposure; Low-level shockwave(s); Low-overpressure event; Low-pressure blast; Low-pressure blast wave; Low-to-moderate shock wave intensities; Mid- to low-intensity explosive blast waves; Mild blast; Mild blast injury; Mild blast overpressure; Mild blast traumatic brain injury (TBI); Mild TBI or subclinical blast exposure; Mild to moderate blast exposure; Moderate blast exposure; Moderate blast levels; Moderate BOP; Moderate level blast-wave; Moderate whole body blast exposure; mTBI or subclinical blasts; Multiple exposures to blast ; Multiple exposures to blast overpressure; Multiple, low intensity blast exposures; No/low-blast exposure; Non-injury level blast exposure; Non-lethal low-level primary blast; Non-lethal penetrating blast exposure; Occupation blast; Occupational blast; Occupational exposure; Occupational exposure to blast; Occupational exposure to impulse noise; Occupational exposure to low-level blast; Occupational history of repetitive blast exposure; Occupational levels of blast overpressure; Occupational levels of low-level blast; Occupational overpressure; Occupational repeated exposure to low-level explosive blast; Open-field low-intensity blast; Operational noise; Overpressure; Overpressure event; Overpressure exposure; Overpressure injury; Overpressure levels; Overpressure levels associated with sub-concussive low-level exposure; Physical shock from ingress strategies used during training; Powerful, low frequency weapons; Pre-clinical blast exposure; Primary blast; Primary blast exposure; Primary blast injury(ies); Primary blast wave; Primary blast wave (or overpressure); Primary blasts by weaponry; Primary lower level blast overpressure (LBOP); Primary low-intensity blast (LIB); Primary moderate blast exposure; Quaternary injuries; Reflected pressure; Relatively low BOP exposures; Repeat low-level blast; Repeat(ed) blast(s); Repeated blast events; Repeated blast exposure; Repeated blast overpressure; Repeated blast overpressure while training; Repeated blast/training exposure; Repeated BOP exposure; Repeated controlled blast exposure; Repeated detonating explosions; Repeated exposure to blast overpressure (BOP); Repeated exposure to low-level blast; Repeated exposure to low-level blast events; Repeated exposure to low-level blast overpressure (BOP); Repeated low intensity blast exposure(s); Repeated low-level blast exposure(s); Repeated low-level blast overpressure exposure; Repeated low-level blast(s); Repeated low-level blast-related head trauma; Repeated low-level BOP exposure; Repeated occupational exposure to sublethal low-level blasts; Repetitive; Repetitive blast exposure; Repetitive exposure from these weapons systems; Repetitive exposure to low-intensity blasts; Repetitive exposure to low-level blast(s); Repetitive low-energy blast exposure; Repetitive low-level blast (LLB); Repetitive low-level blast explosions; Repetitive low-level blast exposure; Repetitive low-level explosive blasts; Repetitive low-level primary blast; Repetitive short term low-level blast; Repetitive sub-concussive or undiagnosed events; Repetitively exposed to low-level explosive blasts ; Routine occupation blast exposure; Routinely and repetitively exposure to low-level blast; Routinely exposed to low-level blast; Secondary blast; Secondary blast injuries; Shock wave exposure; Short-lasting impulse noise; Single blast; Single low-intensity blast exposure; Single, low-intensity blast; Steady state noises; Subclinical blast; Subclinical blast exposure(s); Subclinical blast overpressures; Subclinical exposure; Subclinical impacts and overpressure; Subclinical wave shock exposure; Subconcussive blast exposure; Sub-concussive blast overpressure levels; Sub-concussive repetitive low-level blast exposure; Sublethal blast overpressure; Sublethal BOP levels; Tertiary blast; Tertiary blast injuries; Unprotected occupational exposures to low-level BOP; Weapon operator blast overpressure exposure; Whole-body blast exposure

*Terms used to describe both HLB and LLB.*

acute to subacute effects of blast; blast exposure(s); blast overpressure injury; blast wave(s); blast(s); blasting; blast-related postconcussion syndrome; chronic blast exposure; impulse noise; isolated blast; overpressure exposure; primary blast; primary blast exposure; primary blast injury(ies); repeated blast overpressure; and tertiary blast
